# Seasonal Changes and Relationships in Training Loads, Neuromuscular Performance, and Recovery and Stress State in Competitive Female Soccer Players

**DOI:** 10.3389/fspor.2021.757253

**Published:** 2021-10-11

**Authors:** Ai Ishida, Caleb D. Bazyler, Adam L. Sayers, Michael H. Stone, Jeremy A. Gentles

**Affiliations:** ^1^Department of Sports, Exercise, Recreation, and Kinesiology, East Tennessee State University, Johnson City, TN, United States; ^2^School of Applied Health Sciences and Wellness, Ohio University, Athens, OH, United States; ^3^Center for Global Sport Leadership, East Tennessee State University, Johnson City, TN, United States; ^4^Center of Excellence in Sports Science and Coaching Education, East Tennessee State University, Johnson City, TN, United States

**Keywords:** fatigue, performance, team sport, power, athlete monitoring

## Abstract

**Background:** The purpose of this study was to examine seasonal changes in training load (TL), neuromuscular performance, subjective recovery, and stress state, and to investigate the relationships between acute and chronic TL and neuromuscular performance in competitive female soccer players.

**Methods:** Nine competitive female soccer players (20.0 ± 1.7 years; 60.3 ± 6.3 kg; 164.0 ± 5.8 cm) completed the Short Recovery and Stress Scale and the countermovement jump (CMJ) with polyvinyl chloride pipe (CMJ0) and 20 kg barbell (CMJ20) at 2–3 h before 1st match (NC_1_), 6th match (NC_2_), 9th match (C_1_), and 15th match (C_2_) of the competitive season. TL included total distance, high-speed running, and PlayerLoad. Acute and chronic TL was calculated by using the average of 2 days (D_2_), 7 days (D_7_), and 21 days (D_21_) prior to four different match play.

**Results:** Significant decreases were found from NC_1_ to C_1_ in D_7_ total distance [*p* = 0.03, Cohen's effect size (d_z_) = 1.40]. D_7_ total distance and PlayerLoad significantly decreased from NC to C_1_ and C_2_ (*p* = 0.001–0.01, d_z_ = 1.40–1.72). Significant increases were observed from NC_1_ to C_1_ in CMJ0 jump height (*p* = 0.03, d_z_ = 1.40), (*p* = 0.021, d_z_ = 1.44), and peak power (*p* = 0.03, d_z_ = 1.32). Significant negative correlations were observed for D_7_ total distance and CMJ0 jump height (*p* = 0.02, *r* = 0.79) and peak power (*p* = 0.03, *r* = 0.71) at C_2_, while significant positive correlations were observed at C_1_ for D_7_ PlayerLoad and CMJ0 jump height (*p* = 0.02, *r* = 0.80).

**Conclusion:** Polyvinyl chloride pipe **(**CMJ0) jump height and peak power may increase from preseason to the midcompetitive season. Seasonal variations may affect the relationships between D_7_ TL and CMJ0 performance.

## Introduction

Soccer is an intermittent sport consisting of walking, running, sprinting, and changing of direction, kicking, and heading for 90 min (Stølen et al., 2005). Due to the physical demands of the match, the practitioners (e.g., sports coaches, strength and conditioning coaches, sports scientists) require long-term strategic training plans to gradually accumulate training load (TL) and to develop physical capacity of the players during a pre-season (Stølen et al., 2005). However, it may be difficult to improve the physical capacity of the players during this period for the National Collegiate Athletic Association (NCAA) soccer. The NCAA soccer schedule allows only 2 to 3 weeks of pre-season training followed by 12 to 16 weeks of the competitive season (Sams et al., 2018, 2020; Walker et al., 2019; McFadden et al., 2020). As a result, the NCAA coaching staff often plans higher TLs during pre-season compared to the competitive season (McFadden et al., 2020), which increases the risk of injuries (Agel and Schisel, 2013), psychological stress, and physiological damage (Walker et al., 2019). In addition, the NCAA restricts players from reporting their training to the coaching staff during the summer break (May–July) and evidence indicates that players accumulate limited TL during this period (Sams et al., 2018, 2020; Walker et al., 2019; McFadden et al., 2020). Therefore, the lack of training in the summer often results in a short, high volume pre-season preparation at the NCAA soccer, increasing injuries, and compromising the physical and psychological preparation of the player for the competitive season (Eckard et al., 2018).

Integration of an athlete monitoring program would be useful to develop physical and psychological preparation of the player in NCAA soccer. Athlete monitoring programs primarily aim to maximize sports performance and improve program efficacy while minimize fatigue during the competitive season (Ishida et al., 2021b). Specifically, one aspect of an athlete monitoring program is aimed at quantifying dose-response relationships in order to manage fatigue and to improve training efficacy during the competitive season (Halson, 2014; Gabbett et al., 2017). The monitoring data can inform the practitioners about a current physical and psychological state of the player and assist their decision-making for TL management. Common measures of an athlete monitoring program, particularly for fatigue management, include the Global Navigation Satellite System (GNSS), subjective recovery and stress state, and the countermovement jump (CMJ) (Sams et al., 2018; Travis et al., 2018, 2020; Walker et al., 2019; McFadden et al., 2020; Draper et al., 2021; Ishida et al., 2021c). The GNSS devices have been used to quantify external TL, including the TL of soccer match play, which have been associated with acute muscle damage and the alterations in neuromuscular performance (de Hoyo et al., 2016; Russell et al., 2016; Coppalle et al., 2019; Wiig et al., 2019; Ishida et al., 2021a). Ishida et al. (2021b) reported the strong negative correlations between the changes in loaded CMJ, peak power (PP), and total running distance (*p* = 0.02, *r* = 0.65) at 12 h post-match in the NCAA female soccer players. Therefore, the assessment and manipulation of TL using GNSS can be beneficial for maximizing neuromuscular performance.

Measures of subjective recovery and stress state and CMJ are common monitoring tools to quantify the response to TL. The combined use of these measures with GNSS allows for quantifying a dose–response relationship (Halson, 2014; Impellizzeri et al., 2019; Draper et al., 2021; Ishida et al., 2021b). The Short Recovery and Stress Scale (SRSS) is a reliable questionnaire (Kellmann and Kölling, 2019) and consists of eight subscales including physical performance capability (PPC), mental performance capability (MPC), emotional balance (EB), overall recovery (OR), muscular stress (MS), lack of activation (LA), negative emotional state (NES), and overall stress (OS). Current literature (Wiewelhove et al., 2016; Hitzschke et al., 2017; Pelka et al., 2018; Travis et al., 2020) has shown that the SRSS scales are reflective of acute high TLs. The CMJ is also an easy and reliable measure to assess the neuromuscular performance in female soccer players (Andersson et al., 2008; Nedelec et al., 2014; de Hoyo et al., 2016; Claudino et al., 2017). Current evidence also indicates that CMJ performance alternations can be affected by TL and are associated with neuromuscular and physiological damage (Andersson et al., 2008; de Hoyo et al., 2016; Silva et al., 2018; Hader et al., 2019). For example, Andersson et al. (2008) found that statistically significant decreases were observed in CMJ jump height with substantially increased serum creatine kinase (CK) at 21 h after a soccer match play in the elite female soccer players. Therefore, the SRSS and CMJ measure monitoring subjective and objective responses to TL that may provide a better understanding of the response to the manipulations and variations of TL.

Among the soccer players, seasonal variability of TL, physical capacity, performance, and subjective recovery and stress state should be considered. Several studies (Malone et al., 2015; Clemente et al., 2019, 2020; Nobari et al., 2021b) indicate that TL can be substantially higher during a preseason in soccer than the typical loads during the competitive season. For example, Clemente et al. (2020) reported that the weekly total distance covered was considerably higher during pre-season than the end of the competitive season in the professional male soccer players. As TL tends to decrease, physical performance tends to increase as the competitive season progresses (Dragijsky et al., 2017; Sams et al., 2018; Emmonds et al., 2020). However, seasonal variability could be problematic, particularly for quantifying the relationship between TL and the alterations of neuromuscular performance. Although the meta-analyses (Silva et al., 2018; Hader et al., 2019) showed TL is inversely related to physical performance, no investigations have been performed to quantify the relationship interacting with seasonal variability. This information would provide the practitioners with an understanding of how monitoring measures can be useful for associating neuromuscular performance and subjective recovery status with TL variations.

With respect to the NCAA female soccer season, athlete monitoring measures may be incorporated to appropriately guide physical preparation of the player from the pre-season to the end of the competitive season (Ishida et al., 2021b). Although the seasonal variations in TL and neuromuscular performance in the soccer players would occur (Dragijsky et al., 2017; Sams et al., 2018; Walker et al., 2019; Emmonds et al., 2020), few investigations have been performed as to how measures of neuromuscular performance, subjective recovery, and stress state will change across the competitive season. Therefore, the purpose of this study was to investigate as follows: (1) seasonal changes in TL, neuromuscular performance, and subjective recovery and stress state and (2) to examine the relationship between TL and neuromuscular changes in the division I collegiate female soccer players. It was hypothesized that: (1) TL, neuromuscular performance, and subjective recovery and stress state would vary and (2) negative relationships would be observed between TL and neuromuscular performance across the competitive season in the Division I collegiate female soccer players.

## Materials and Methods

### Design

Data collection occurred throughout the 2019 NCAA soccer season consisting of 2 weeks of pre-season and 11 weeks of the competitive season, respectively. Data were collected by using a GNSS for TL. The average of 2 days (D_2_), 7 days (D_7_), and 21 days (D_21_) GNSS TL was calculated prior to four different match play: 1st match of the competitive season (NC_1_; 1st non-conference match play), 6th match-play of the competitive season (NC_2_; 6th non-conference match play), 9th match of the competitive season (C_1_; 1st conference match play), and 15th match of the competitive season (C_2_; 6th conference match play). Neuromuscular performance and subjective recovery state were evaluated *via* the CMJ and SRSS assessments at 3 h prior to NC_1_, NC_2_, C_1_, and C_2_. Data collection at NC_1_, NC_2_, C_1_, and C_2_ were performed at the first match-play of each week.

### Subjects

In this study, the NCAA division I nine female soccer players were included [age 20.0 ± 1.7 years (age range: 18–22 years); body mass 60.3 ± 6.3 kg; height 164.0 ± 5.8 cm; resistance training experience 1–4 years]. Demographic information was collected on the first day of the pre-season. The inclusion criteria for this study were as follows: (a) players were outfield players (defender, midfielder, or forward) and (b) must have completed all testing sessions. This study included six starters (played 74 to 100% of match time across all the NCAA matches) and three non-starters (played 28 to 44% of match time across all the NCAA matches). Six players were excluded from this study because they could not complete CMJ tests. Depending upon the travel schedule, the players completed 0 to 1 strength maintenance weight training sessions each week during pre-season and non-conference play (day 1–51). Weight training was performed two times per week during the conference play (day 52–82). During this study, all the participants were informed of the risks and benefits and testing procedures before participation. The participants signed informed consent and this study was approved by the University Institutional Review Board.

### Training Load Measure

D_2_, D_7_, and D_21_ TLs were measured by using a GNSS (10 Hz) and accelerometry (triaxial; 100 Hz) units (Catapult OptimEye S5, Catapult Innovations, Team Sport 5.0, Melbourne, Australia). A team of sports scientists powered all the units at least 10 min before the on-field warm-up. Players wore the GNSS units in a vest, which positioned the unit between the shoulder blades. Training and match-derived TL data included the warm-up until the end of the session. Variables of interest related to training volume included total distance (m), high-speed running distance (HSR; m), and PlayerLoad (au). HSR was defined as running above 15 km h^−1^. PlayerLoad was calculated as the square root of the sum of the squared differences of acceleration in all the three axes divided by the device sampling frequency of 100 Hz (Nicolella et al., 2018). According to the previous literature (Scott et al., 2016; Nicolella et al., 2018; Nikolaidis et al., 2018), a 10-Hz GNSS unit demonstrates good-to-moderate reliability for total distance [coefficient of variation (CV) = 1.9%] and running involving accelerations (CV = 1.9–4.3%) and PlayerLoad (CV = 0.0–3.0% in anterior-posterior, medial-lateral, and vertical axes). The average TL of 2, 7, and 21 days prior to an average match play was calculated (Carey et al., 2017; Sams et al., 2018).

### Short Recovery and Stress Scale

The player recovery and stress state were used to assess the subjective recovery and stress state across the 10 week competitive season *via* the SRSS. The players completed the SRSS from an online-based application (Google Forms, Google, California, United States). All the players were fully familiarized with the procedures using a pre-season match. Prior to the SRSS, hydration status was assessed using a refractometer (ATAGO Corporation Limited, Tokyo, Japan) before the SRSS measurement. The players were considered as hydrated if specific gravity of urine was <1.020. The SRSS is rated by using a seven-point Likert scale from 0 (does not fully apply) to 6 (fully applies) and consists of eight subscales including PPC, MPC, EB, OR, MS, LA, NES, and OS. The Recovery Scale (RS) includes PPC, MPC, EB, and OR, while the Stress Scale (SS) includes MS, LA, NES, and OS. The SRSS has shown acceptable internal reliability (α = 0.74 and α = 0.78) (Kellmann and Kölling, 2019).

### Countermovement Jump

The players completed a standardized dynamic warm-up followed by submaximal CMJs at 75% and 100% of perceived maximal efforts, respectively. The players then performed three maximal CMJ trials with a polyvinyl chloride pipe (CMJ0) and with 20 kg barbell (CMJ20) on dual portable force plates (PASPORT Force Platform, PASCO, California, United States of America) by using a sampling frequency of 1,000 Hz. Each load was held across the back on the shoulders. For the CMJ tests, the players stood still on the force plates for at least 1 s and then vertically jumped after flexing the hip, knee, and ankle joints on the command of “3, 2, 1, jump!”. Approximately, a 1-min interval was provided between the CMJ0 and CMJ20 trials. After CMJ testing, the raw data were converted into a comma-separated values file and then analyzed by using a Microsoft Excel sheet (Microsoft Excel, Microsoft, Washington, United States of America) (Chavda et al., 2018). The mean of two trials with the best jump heights (JHs) was used for analysis. Body mass (BM; kg), JH from impulse (cm), modified reactive index (RSI; m s^−1^), peak force (PF; N), relative PF (RPF; N kg^−1^), eccentric impulse (EI; N s^−1^), concentric impulse (CI; N s^−1^), (PP; W), relative PP (RPP; W kg^−1^), eccentric peak power (EPP; W), and concentric peak power (CPP; W) were included as variables of interest. The test-retest reliability of the variables was acceptable in CMJ0 [CV = 2.1–5.9%; intraclass correlation coefficient (ICC) = 0.86–0.97] and CMJ20 (CV = 2.7–6.2%; ICC = 0.76–0.92).

### Statistical Analysis

All the statistical procedures were performed by using the statistical software RStudio (version 1.1.463) and the packages dplyr (0.8.5), rstatix (0.4.0), and stats (3.5.3). One-way repeated analysis of variance was conducted to examine the difference in the CMJ0 and CMJ20 kinetic variables and the TLs across the four different periods (NC_1_, NC_2_, C_1_, and C_2_). For the SRSS and the CMJ variables (JH in CMJ0 and JH and PF in CMJ20) that did not meet the assumption of normality, the Friedman test was performed to identify the differences between the periods. When necessary, *post-hoc* testing with the Bonferroni correction was performed. A Cohen's d_z_ effect sizes (d_z_) were also calculated by using standardized mean difference and were classified as follows; d_z_ < 0.2 = trivial, 0.2 ≤ d_z_ < 0.6 = small, 0.6 ≤ d_z_ < 1.2 = moderate, 1.2 ≤ d_z_ < 2.0 = large, and d_z_ ≥ 2.0 = very large (Hopkins et al., 2009). The Pearson coefficient correlation tests were also conducted to examine the relationship between TL and the changes from NC_1_ to NC_2_, C_1_, and C_2_ in the selected CMJ0 and CMJ20 kinetic variables (JH, RSI, PF, and PP). The correlation coefficient magnitudes were determined by using Hopkin's classification (Hopkins et al., 2009) and were classified as follows: *r* < 0.10 = trivial, 0.10 ≤ *r* < 0.30 = small, 0.30 ≤ *r* < 0.50 = moderate, 0.5 ≤ *r* < 0.7 = large, 0.70 ≤ *r* < 0.90 = very large, 0.90 ≤ *r* < 1.00 = nearly perfect, and 1.0 = perfect. All the data were expressed as mean ± SD. Statistical significance was set at *p* ≤ 0.05.

## Results

### Seasonal Changes in Training Loads, Countermovement Jump, Short Recovery, and Stress State

Statistically significant differences were observed between NC_1_ and C_1_ in D_2_ total distance (*p* = 0.02, d_z_ = 1.53) and PlayerLoad (*p* = 0.05, d_z_ = 1.24). D_2_ total distance and PlayerLoad were also statistically decreased from NC_2_ to C_1_ (total distance, *p* < 0.001, d_z_ = 2.68; PlayerLoad, *p* = 0.04, d_z_ = 1.28) and C_2_ (total distance, *p* = 0.05, d_z_ = 1.86; PlayerLoad, *p* = 0.03, d_z_ = 1.35). Additionally, statistically significant differences were found from NC_1_ to C_1_ in D_7_ total distance (*p* = 0.03, d_z_ = 1.40). D_7_ total distance and PlayerLoad were statistically decreased from NC_2_ to C_1_ (total distance, *p* = 0.03, ES = 1.40; PlayerLoad, *p* = 0.02, d_z_ = 1.72) and C_2_ (total distance, *p* = 0.03, d_z_ = 1.40; PlayerLoad, *p* = 0.02, d_z_ = 1.49) (Table 1). However, no statistical differences were noted for D_21_ total distance, HSR, and PlayerLoad (*p* > 0.05).

**Table 1:** Seasonal changes in training loads.

	**Periods**				**Percentage changes (** ***d***_**z**_ **)**		
**Variables**	**NC_**1**_**	**NC_**2**_**	**C_**1**_**	**C_**2**_**	**NC_**1**_-NC_**2**_**	**NC_**1**_-C_**1**_**	**NC_**1**_-C_**2**_**
**D_**2**_**							
Total distance (m)	4,108.6 ± 1,026.7	3,573.1 ± 365.1	2,638.1 ± 247.1^*^^§^	3,627.8 ± 696.5^§^^¶^	23.9 ± 19.3 (ES = 0.54)	31.9 ± 18.8 (ES = 1.53)	8.7 ± 12.0 (ES = 0.58)
HSR (m)	331.4 ± 295.4	225.9 ± 116.2	144.8 ± 68.0^§^	250.2 ± 128.0	34.8 ± 34.1 (ES = 0.48)	32.2 ± 88.1 (ES = 0.83)	25.9 ± 33.1 (ES = 0.29)
Total PlayerLoad (au)	491.1 ± 129.1	408.8 ± 64.0	361.9 ± 68.8^*^^§^	442.9 ± 110.6^¶^	10.8 ± 17.1 (ES = 0.73)	23.3 ± 20.8 (ES = 1.24)	7.2 ± 10.7 (ES = 0.43)
**D**_**7**_							
Total distance (m)	4,451.9 ± 775.2	5,831.6 ± 1,534.2	3,567.7 ± 317.0^*^^§^	4,164.9 ± 1,744.2^§^	50.6 ± 13.7 (ES = 1.00)	18.0 ± 40.0 (ES = 1.40)	5.1 ± 33.4 (ES = 0.17)
HSR (m)	394.6 ± 180.2	416.4 ± 213.5	318.9 ± 121.1	282.6 ± 226.0	20.9 ± 24.1 (ES = 0.16)	12.3 ± 43.8 (ES = 0.75)	25.2 ± 31.7(ES = 0.55)
Total PlayerLoad (au)	520.5 ± 104.8	638.8 ± 168.0	464.5 ± 62.4^§^	471.8 ± 181.1	33.5 ± 10.5 (ES = 0.83)	9.2 ± 35.3 (ES = 1.03)	7.9 ± 26.2 (ES = 0.28)
**D**_**21**_							
Total distance (m)	4,524.9 ± 713.3	5,063.4 ± 1,348.1	4,692.4 ± 1,173.8	4,717.1 ± 1,671.6	7.9 ± 32.8 (ES = 0.41)	6.0 ± 49.2 (ES = 0.14)	8.2 ± 8.5 (ES = 0.11)
Total PlayerLoad (au)	527 ± 101.6	561.6 ± 140.4	537.9 ± 138.3	532.7 ± 140.4	4.4 ± 30.0 (ES = 0.26)	4.4 ± 43.9 (ES = 0.08)	5.0 ± 7.3 (ES = 0.03)
HSR (m)	318.6 ± 139.1	365.9 ± 191.4	372.7 ± 168.7	311.5 ± 192.1	4.4 ± 22.5 (ES = 0.54)	17.2 ± 43.9 (ES = 0.72)	0.5 ± 14.5 (ES = 0.05)

*d_z_, Cohen's d_z_ effect size; NC_1_, 1st non-conference match-play; NC_2_, 7th non-conference match-play; C_1_, 1st conference match-play; C_2_, 6th conference match-play; HSR, high speed running distance*.

* *Denotes p ≤ 0.05 from NC_1_;*

§ *denotes p ≤ 0.05 from NC_2_;*

¶ *denotes p ≤ 0.05 from C_1_*.

**Figure 1:**
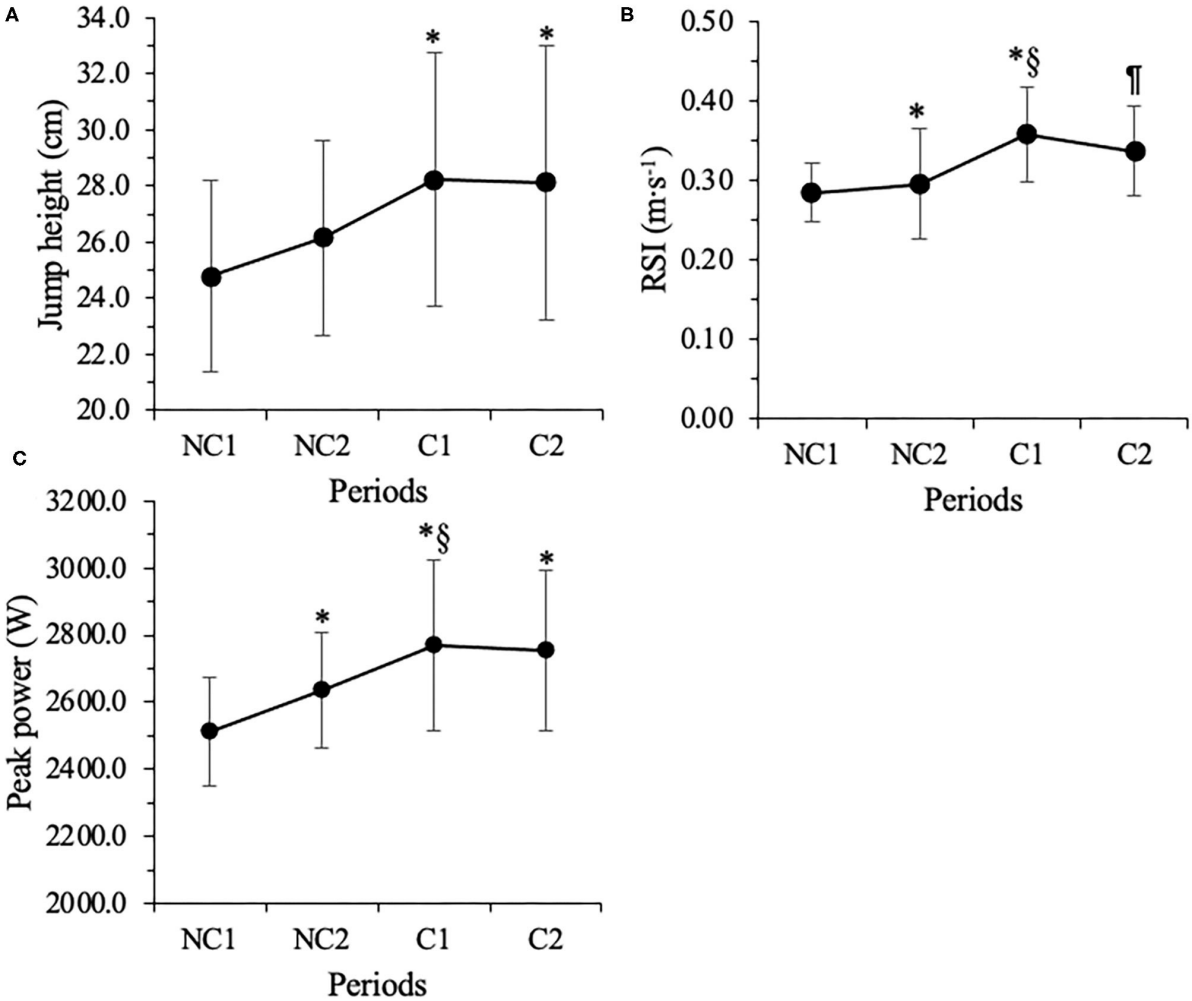
Seasonal changes in unloaded countermovement jump height **(A)**, modified strength index (RSI) **(B)**, and peak power **(C)**. NC_1_ = 1st non-conference match-play. NC_2_ = 7th non-conference match-play. C_1_ = 1st conference match-play. C_2_ = 6th conference match-play. *Denotes *p* ≤ 0.05 from NC_1_. ^§^Denotes *p* ≤ 0.05 from NC. ^¶^Denotes *p* ≤ 0.05 from C_1_.

Statistically significant differences were observed from NC_1_ to C_1_ in CMJ0 JH (*p* = 0.03, d_z_ = 1.40), RSI (*p* = 0.02, d_z_ = 1.44), PP (*p* = 0.034, d_z_ = 1.32), and CAP (*p* = 0.01, d_z_ = 1.74) (Figure 1). Statistically significant differences were also noted from NC_1_ to C_2_ for CMJ0 JH (*p* = 0.015, d_z_ = 1.53), PP (*p* = 0.01, d_z_ =1.57), and RPP (*p* = 0.03, d_z_ =1.40). Additionally, CMJ20 JH, PP, and RPP showed statistically significant differences from NC_1_ to C_2_ (JH, *p* = 0.019, d_z_ = 1.47; PP, *p* = 0.03, d_z_ = 1.37; RPP, *p* = 0.02, d_z_ = 1.49). However, no statistically significant changes were seen in BM and CMJ0 and CMJ20 PF, RPF, and EAP (*p* > 0.05) (Table 2). No significant changes were observed in any of the SRSS items across time (*p* > 0.05).

**Table 2:** Seasonal changes in unloaded and loaded countermovement jump kinetics.

					**Percentage changes (d**_**z**_ **)**		
**Variables**	**NC_**1**_**	**NC_**2**_**	**C_**1**_**	**C_**2**_**	**NC_**1**_-NC_**2**_**	**NC_**1**_-C_**1**_**	**NC_**1**_-C_**2**_**
**CMJ0**							
BW	62.2 ± 5.9	62.5 ± 6.1	62.5 ± 6.3	61.7 ± 5.7	0.2 ± 1.4 (ES = 0.29)	0.3 ± 1.5 (ES = 0.24)	0.7 ± 3.2 (ES = 0.26)
JH (cm)	24.8 ± 3.4	26.1 ± 3.5	28.2 ± 4.5^*^	28.1 ± 4.9^*^	8.3 ± 8.0 (ES = 0.73)	14.1 ± 11.4 (ES = 0.89)	13.3 ± 9.6 (ES = 0.85)
RSI (m·s^−1^)	0.28 ± 0.04	0.30 ± 0.07	0.36 ± 0.06^*^^§^	0.34 ± 0.06	22.1 ± 15.1 (ES = 0.18)	26.6 ± 21.1 (ES = 1.44)	20.8 ± 29.8 (ES = 0.72)
PF (N)	734.1 ± 76.2	779.7 ± 152.8	829.7 ± 101.4	743.1 ± 78.6	7.6 ± 10.9 (ES = 0.46)	13.4 ± 11.5 (ES = 1.22)	1.9 ± 12.9 (ES = 0.11)
RPF (N·kg^−1^)	11.9 ± 1.4	12.6 ± 2.8	13.5 ± 2.5	12.1 ± 1.5	7.8 ± 11.2 (ES = 0.45)	13.1 ± 12.2 (ES = 1.10)	2.6 ± 13.1 (ES = 0.18)
EI (N·s^−1^)	73.5 ± 11.8	73.6 ± 11.2	77.5 ± 11.5	77.7 ± 9.7	5.6 ± 6.4 (ES = 0.03)	6.2 ± 13.3 (ES = 0.47)	6.8 ± 13.3 (ES = 0.53)
CI (N·s^−1^)	139.7 ± 12.5	143.8 ± 14.0	148.7 ± 13.0	145.1 ± 9.7	3.6 ± 5.2 (ES = 0.54)	6.6 ± 6.8 (ES = 1.03)	4.0 ± 7.4 (ES = 0.56)
PP (W)	2511.7 ± 162.9	2637.3 ± 171.0^*^	2769.4 ± 255.6^*^	2752.3 ± 238.1^*^	5.2 ± 5.4 (ES = 1.30)	10.3 ± 8.2 (ES = 1.32)	9.6 ± 6.5 (ES = 1.57)
RPP (W·kg^−1^)	40.6 ± 3.9	42.4 ± 3.7	44.5 ± 5.5^*^	44.8 ± 4.8	5.0 ± 5.4 (ES = 0.98)	9.6 ± 8.6 (ES = 1.23)	10.4 ± 6.6 (ES = 1.74)
EAP (W)	345.7 ± 91.0	330.1 ± 97.4	370.2 ± 65.3	353.5 ± 55.0	13.6 ± 17.2 (ES = 0.20)	11.4 ± 25.7 (ES = 0.39)	8.3 ± 35.1 (ES = 0.11)
CAP (W)	1308.3 ± 126.2	1378.5 ± 155.2	1462.8 ± 155.8^*^^§^	1412.0 ± 132.2	6.6 ± 5.9 (ES = 0.91)	12.0 ± 7.8 (ES = 1.64)	8.3 ± 9.2 (ES = 1.04)
**CMJ20**							
JH (cm)	16.4 ± 2.1	17.0 ± 2.0	18.5 ± 2.1	18.5 ± 2.7^*^	5.5 ± 13.1 (ES = 0.61)	14.2 ± 18.0 (ES = 0.81)	13.3 ± 10.7 (ES = 0.85)
RSI (m·s^−1^)	0.18 ± 0.03	0.18 ± 0.04	0.21 ± 0.04	0.19 ± 0.03	12.3 ± 11.0 (ES = 0.13)	14.3 ± 18.4 (ES = 0.85)	8.9 ± 23.3 (ES = 0.34)
PF (N)	684.3 ± 83.3	714.1 ± 132.6	798.2 ± 130.1	702.5 ± 50.4	13.2 ± 17.3(ES = 0.38)	17.3 ± 18.2 (ES = 0.77)	3.8 ± 13.1 (ES = 0.14)
RPF (N·kg^−1^)	8.4 ± 1.1	8.7 ± 1.8	9.8 ± 2.1	8.7 ± 0.9	13.4 ± 17.9 (ES = 0.36)	17.1 ± 18.7 (ES = 0.96)	4.6 ± 13.2 (ES = 0.33)
EI (N·s^−1^)	86.5 ± 11.6	88.9 ± 11.8	89.5 ± 9.9	90.2 ± 9.3	0.9 ± 6.5 (ES = 0.35)	4.1 ± 8.0 (ES = 0.59)	5.6 ± 15.6 (ES = 0.38)
CI (N·s^−1^)	150.3 ± 18.3	157.7 ± 17.4^*^	160.5 ± 13.1	156.3 ± 14.4	2.4 ± 6.6 (ES = 1.54)	7.5 ± 9.3 (ES = 0.79)	4.3 ± 5.5 (ES = 0.74)
PP (W)	2,483.1 ± 185.9	2,596.7 ± 188.8	2,696.4 ± 232.5	2,667.8 ± 173.4^*^	4.3 ± 7.5 (ES = 1.14)	8.9 ± 10.4 (ES = 0.92)	7.7 ± 6.2 (ES = 1.37)
RPP (W·kg^−1^)	30.4 ± 2.5	31.7 ± 2.1	32.9 ± 2.9	33.0 ± 2.5^*^	4.3 ± 8.1 (ES = 0.91)	8.6 ± 11.2 (ES = 0.84)	8.5 ± 6.6 (ES = 1.49)
EAP (W)	463.6 ± 92.2	446.9 ± 118.1	467.5 ± 100.1	441.6 ± 77.6	5.1 ± 14.7 (ES = 0.31)	0.8 ± 8.0 (ES = 0.11)	3.8 ± 11.8 (ES = 0.50)
CAP (W)	1,215.4 ± 153.5	1,275.6 ± 180.5	1,332.8 ± 198.1	1,286.8 ± 124.5	4.9 ± 7.6 (ES = 0.65)	9.9 ± 10.7 (ES = 0.92)	6.6 ± 9.8 (ES = 0.73)

*d_z_, Cohen's d_z_ effect size; NC_1_, 1st non-conference match-play; NC_2_, 7th non-conference match-play; C_1_, 1st conference match-play; C_2_, 6th conference match-play; CMJ0, countermovement jump with a polyvinyl chloride pipe; CMJ20, countermovement jump with a 20-kg barbell; BW, body weight; JH, jump height; RSI, modified reactive strength index; PF, peak force; RPF, relative PF; EI, eccentric impulse; CI, concentric impulse; PP, peak power; RPP, relative PP; EAP, eccentric average power; CAP, concentric average power*.

* *denotes p ≤ 0.05 from NC_1_ and*

§ *denotes p ≤ 0.05 from NC_2_*.

### Seasonal Relationships Between Training Loads and the Changes in Countermovement Jump Performance

**Figure 2:**
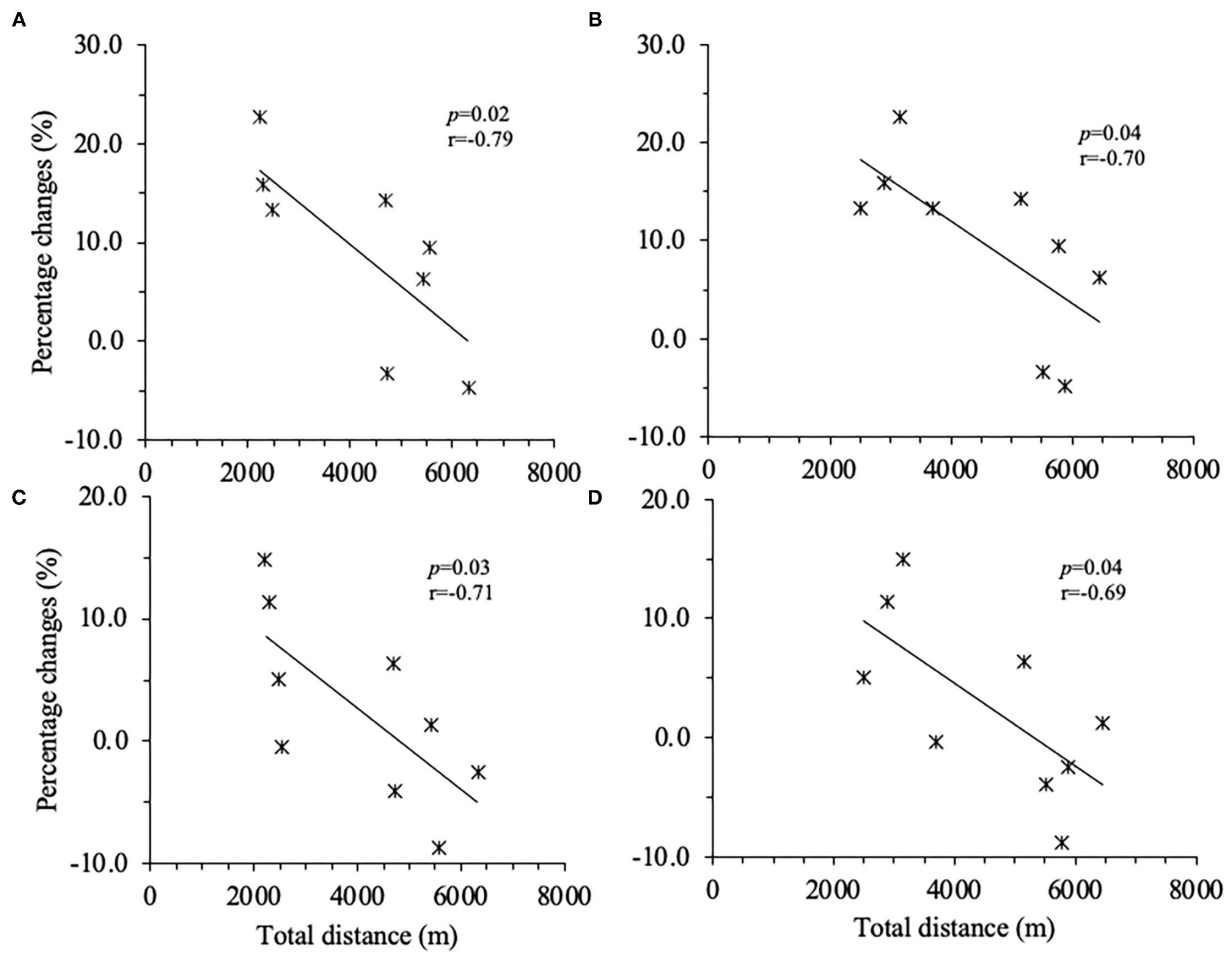
Correlations between percentage changes from 1st non-conference match-play to 6th conference match-play inunloaded countermovement jump (CMJO) jump height and peak power and total distance. **(A)** Between jump height and D_7_ total distance, **(B)** between jump height and D_21_ total distance, **(C)** peak power and D_7_ total distance, and **(D)** peak power and D_21_ total distance. D_7_ = average of 7 days. D_21_ = average of 21 days.

Very large positive correlations were observed at C_1_ between D_2_ total PlayerLoad and the changes from NC_1_ to C_1_ in CMJ0 JH (*p* = 0.05, *r* = 0.67); D_7_ total PlayerLoad and CMJ0 JH (*p* = 0.02, *r* = 0.80), RSI (*p* = 0.03, *r* = 0.80), and PF (*p* = 0.02, *r* = 0.77). In CMJ20, very large positive correlations were also found between D_2_ total PlayerLoad and the changes from NC_1_ to C_1_ in CMJ0 JH (*p* < 0.001, *r* = 0.88), RSI (*p* = 0.001, *r* = 0.86), and PF (*p* = 0.001, *r* = 0.93); D_7_ total PlayerLoad and JH (*p* = 0.01, *r* = 0.87) and PF (*p* < 0.001, *r* = 0.90). At C_2_, very large negative correlations were observed between D_7_ total distance and the changes from NC_1_ to C_2_ CMJ0 JH (*p* = 0.02, *r* = 0.79) and PP (*p* = 0.03, *r* = 0.71); D_21_ total distance and CMJ0 JH (*p* = 0.04, *r* = 0.70) and PP (*p* = 0.04, *r* = 0.69) (Figure 2).

## Discussion

The purposes of this study were to investigate: (1) seasonal changes in TL, neuromuscular performance, subjective recovery, and stress state and (2) the relationships between TL and neuromuscular changes in division I collegiate female soccer players. The main findings of this study were: (1) D_7_ total distance and PlayerLoad were lower at the conference period than the pre-season and non-conference periods, (2) CMJ0 JH, PP, and RSI were statistically higher at C_1_ than NC_1_, (3) positive correlations were found between D_7_ PlayerLoad and unloaded CMJ JH, PP, and PF at C_1_ while negative correlations were observed between D_2_ and D_7_ total distance and CMJ0 JH and PP at C_2_. This study showed that neuromuscular performance gradually increased from the pre-season to the conference play and there was a negative relationship between D_7_ TL and CMJ at C_2_.

In professional and NCAA soccer, a pre-season is considered as a physical preparatory phase to develop physical capabilities for 2–4 weeks prior to the competitive season. However, practitioners are aware that the length of the pre-season would not be sufficient to adequately improve characteristics of fitness (Dragijsky et al., 2017; Sams et al., 2018; Emmonds et al., 2020; McFadden et al., 2020). The short pre-season may also not provide sufficient recovery time between training sessions, potentially resulting in greater physiological and psychological damage (McFadden et al., 2020; Nobari et al., 2021a,b). Our data indicate that D_7_ total distance and PlayerLoad at C_1_ were statistically lower than NC_1_ and NC_2_. This agrees with current evidence that soccer players will typically accumulate higher TLs during the pre-season and the early competitive season (Malone et al., 2015; Clemente et al., 2019, 2020). For example, Malone et al. (2015) found that total distance was substantially higher during the early competitive season (weeks 7–12) compared to the end of the competitive season (weeks 37–42) in elite English male soccer players (*p* < 0.05, ES = 0.84). Therefore, similar to previous investigations (Malone et al., 2015; Clemente et al., 2019, 2020), TL was highest during the pre-season and the early competitive season in the Division I NCAA female soccer players. However, the NCAA female soccer players may not accumulate sufficient TL after the summer break (May–July). Therefore, practitioners may need to manipulate and progress from the pre to the early competitive season to minimize the risk of injuries.

CMJ0 JH, RSI, and PP statistically increased from NC_1_ to C_1_, although weight training frequency was inconsistent and limited from NC_1_ to C_1_. Similar to our findings, several studies (Dragijsky et al., 2017; Sams et al., 2018; Emmonds et al., 2020) have shown that neuromuscular performance improves among soccer players from pre-season to the mid or end of the competitive season. Sams et al. (2018) reported that squat JH showed a moderate increase from baseline to the 8th match of the competitive season (*p* = 0.039, ES = 1.01) in the NCAA Division I female soccer players. Improved neuromuscular performance at the mid and end of the competitive season may be explained by the training status of the players prior to the NCAA pre-season. The NCAA prohibits division I soccer teams from starting pre-season until 2–3 weeks prior to the first competition (National Collegiate Athletic National Collegiate Athlete Association, 2021). Nonetheless, the NCAA also restricts strength and conditioning coaches from having mandatory physical training sessions and monitoring their TL during the summer period (12–14 weeks from May to early August). The NCAA restrictions could be detrimental for reasonable TL maintenance or accumulation during the summer and can result in a sudden increase in TL at pre-season, increasing the risk of injuries, muscle damage, and autonomic nervous system fatigue (Agel and Schisel, 2013; Walker et al., 2019; Sekiguchi et al., 2021). The 2–3 weeks of pre-season, as a result of the NCAA regulation, may leave players physically underprepared during the non-conference play and the 12–16 weeks of the competitive season.

The correlations between D_7_ TL and CMJ0 changes were positive at C_1_, while very large negative correlations were observed between D_7_ total distance and CMJ0 JH and PP at C_2_. These findings may indicate that the relationship between TL and neuromuscular performance may be altered across the competitive season. Our finding at C_2_ agrees with the numerous previous findings (Andersson et al., 2008; Silva et al., 2018; Hader et al., 2019) indicating that jump performance can be negatively affected both acutely and chronically by training. For example, a meta-analysis by Hader et al. (2019) reported that a large negative correlation was found between high-speed running distance (>19.8 km/h) and the CMJ0 PP (*r* = 0.52, 95% CI 0.64–0.40) at 24 h post-match. However, the correlations between D_7_ TL and CMJ0 changes were positive at C_1_. The disagreement between our finding and previous literature (Andersson et al., 2008; Silva et al., 2018; Hader et al., 2019) may be explained by the training status of the player. The NCAA soccer players may be in physically undertrained status due to the lack of insufficient training volume prior to the pre-season. When prescribing intense/high volume during 2–3 weeks of a pre-season, the training prescription may increase the neuromuscular performance of the player from the pre-season to the mid and late competitive season (Dragijsky et al., 2017; Sams et al., 2018; Emmonds et al., 2020) resulting in the positive correlations between D_7_ TL and CMJ0 changes from NC_1_ to C_1_. Based on our findings and previous literature (Dragijsky et al., 2017; Sams et al., 2018; Emmonds et al., 2020), the effects of seasonal variations may affect the assessment of neuromuscular performance in relation to TL in NCAA soccer.

## Limitations

There are three main limitations of this study. First, the sample size of this study was limited. The data were collected as a part of ongoing athlete monitoring, so this study could not maintain large sample sizes due to injuries affecting jump testing. Second, there were no measures of additional physical performance abilities such as maximum strength, sprinting, change of directions, and intermittent endurance performance over the period. Third, no internal TL measures were included in this study. Future investigation should include other performance tests and examine the effects of weight training sessions with larger sample sizes.

### Practical Application

In the NCAA Division I female soccer players, CMJ0 and CMJ20 may increase from the first match play to the midcompetitive and late season. Practitioners (e.g., sports coaches, strength and conditioning coaches, sports scientists) should be aware that longer-term strategic training plans may be required to develop and maximize neuromuscular performance at the end of the competitive season. The practitioners should also carefully impart to collegiate athletes the importance of the quantification of the summer and pre-season TL for maximizing neuromuscular performance at the early competitive season. In addition, care should also be taken when analyzing and interpreting the relationship between acute TL and CMJ0 performance due to the seasonal variations associated with physical preparedness of the player. Our findings demonstrate that CMJs may be a worthwhile test to quantify neuromuscular alternations at the mid and end of the competitive season.

## Data Availability Statement

The raw data supporting the conclusions of this article will be made available by the authors, without undue reservation.

## Ethics Statement

The studies involving human participants were reviewed and approved by East Tennesse State University. The patients/participants provided their written informed consent to participate in this study.

## Author Contributions

AI, CB, AS, MS, and JG contributed to study design and implementation. AI and JG carried out all the data collection and analysis. All authors contributed to data analysis, interpretation, discussion of the results, editing and reviewing of the article, and read and agreed to the final version of the submitted manuscript.

## Conflict of Interest

The authors declare that the research was conducted in the absence of any commercial or financial relationships that could be construed as a potential conflict of interest.

## Publisher's Note

All claims expressed in this article are solely those of the authors and do not necessarily represent those of their affiliated organizations, or those of the publisher, the editors and the reviewers. Any product that may be evaluated in this article, or claim that may be made by its manufacturer, is not guaranteed or endorsed by the publisher.
