# Supplementary material for: Seasonal Changes and Relationships in Training Loads, Neuromuscular Performance, and Recovery and Stress State in Competitive Female Soccer Players
Source: Front Sports Act Living. 2021 Oct 11;3:757253. doi: 10.3389/fspor.2021.757253 (PMC8542871; doi:10.3389/fspor.2021.757253)
Supplement: Supplementary file 1 [file Table_1.DOCX]

| Suppl Table 1. Correlations between training loads and changes in selected unloaded countermovement jump variables across time points | | | | | | |
| --- | --- | --- | --- | --- | --- | --- |
|  | **Total distance** | | | **Total PlayerLoad** | | |
| **Variables** | **NC_1_–NC_2_** | **NC_1_–C_1_** | **NC_1_–C_2_** | **NC_1_–NC_2_** | **NC_1_–C_1_** | **NC_1_–C_2_** |
| D_2_ |  |  |  |  |  |  |
| JH | 0.67*(0.02,0.92) | 0.57 (-0.15,0.89) | -0.55 (-0.89,0.18) | 0.53 (-0.10,0.86) | 0.86*(0.39,0.97) | -0.30 (-0.81,0.45) |
| RSI | 0.14 (-0.57,0.74) | 0.49 (-0.25,0.87) | -0.40 (-0.84,0.35) | 0.20 (-0.45,0.72) | 0.67 (-0.06,0.93) | -0.28 (-0.79,0.48) |
| PF | 0.34 (-0.42,0.82) | 0.51 (-0.23,0.89) | -0.06 (-0.70,0.63) | 0.54 (-0.09,0.86) | 0.64 (-0.12,0.93) | 0.12 (-0.59,0.72) |
| PP | 0.34 (-0.42,0.82) | 0.56 (-0.16,0.89) | -0.49 (-0.87,0.26) | 0.50 (-0.14,0.85) | 0.73 (0.06,0.95) | -0.31 (-0.81,0.45) |
| D_7_ |  |  |  |  |  |  |
| JH | 0.44 (-0.31,0.86) | 0.50 (-0.24,0.87) | -0.76*(-0.93,-0.20) | 0.45 (-0.22,0.83) | 0.80*(0.22,0.96) | -0.60 (-0.90,0.11) |
| RSI | 0.20(-0.54,0.76) | 0.56 (-0.16,0.89) | -0.52 (-0.88,0.21) | 0.32 (-0.35,0.77) | 0.77*(0.14,0.96) | -0.50(-0.86,0.31) |
| PF | -0.21 (-0.77,0.53) | 0.74*(0.15,0.94) | -0.36 (-0.83,-0.40) | 0.25 (-0.41,0.74) | 0.77*(0.15,0.96) | -0.23 (-0.77,0.51) |
| PP | -0.25 (-0.78,0.50) | 0.45 (-0.30,0.86) | -0.71*(-0.93,-0.09) | -0.15 (-0.69,0.49) | 0.70*(-0.01,0.94) | -0.63 (-0.91,0.06) |
| D_21_ |  |  |  |  |  |  |
| JH | 0.51 (-0.23,0.88) | 0.27 (-0.48,0.79) | -0.70*(-0.93,-0.07) | 0.49 (-0.16,0.84) | 0.67*(0.06,0.93) | -0.50 (-0.88,0.24) |
| RSI | 0.16 (-0.57,0.74) | 0.09 (-0.61,0.71) | -0.44 (-0.86,0.31) | 0.34 (-0.33,0.78) | 0.41 (-0.42,0.86) | -0.36 (-0.83,0.40) |
| PF | -0.01 (-0.67,0.66) | -0.08 (-0.70,0.62) | -0.37 (-0.83,0.39) | 0.41 (-0.26,0.81) | 0.31 (-0.50,0.83) | -0.22 (-0.77,0.52) |
| PP | -0.07 (-0.70,0.62) | 0.05 (-0.63,0.69) | -0.69*(-0.93,-0.24) | -0.04 (-0.62,0.42) | 0.41 (-0.42,0.86) | -0.60 (-0.90,0.11) |
| Note. NC_1_=1st non-conference play. NC_2_=6th non-conference play. C_1_=1st conference-play. C_2_= 6th conference-play. D_2_= 2-day average. D_7_=7-day average. D_21_=21-day average. JH=Jump height. RSI=Modified reactive strength index. PF=Peak force. PP=Peak power. *=denotes *p*≤0.05 | | | | | | |
|  |  |  |  |  |  |  |

| Suppl Table 2. Correlations between training loads and changes in selected loaded countermovement jump variables across time points | | | | | | |
| --- | --- | --- | --- | --- | --- | --- |
|  | **Total distance** | | | **Total PlayerLoad** | | |
| **Variables** | **NC_1_–NC_2_** | **NC_1_–C_1_** | **NC_1_–C2** | **NC_1_–NC_2_** | **NC_1_–C_1_** | **NC_1_–C2** |
| D_2_ |  |  |  |  |  |  |
| JH | 0.53 (-0.21,0.88) | 0.59 (-0.12,0.90) | -0.24 (-0.78,0.50) | 0.60*(0.00,0.88) | 0.88*(0.36,0.98) | -0.01 (-0.67,0.66) |
| RSI | 0.50 (-0.25,0.87) | 0.58 (-0.14,0.90) | -0.58 (-0.90,-0.13) | 0.20 (-0.45,0.72) | 0.86*(0.40,0.97) | -0.67*(-0.92,-0.01) |
| PF | 0.60 (-0.10,0.90) | 0.64 (-0.05,0.91) | -0.59 (-0.90,-0.12) | 0.51(-0.13,0.85) | 0.93*(0.65,0.99) | -0.61(-0.91,0.10) |
| PP | -0.38 (-0.83,0.38) | -0.41 (-0.84,0.35) | -0.51 (-0.88,0.23) | -0.27 (-0.62,0.58) | -0.16 (-0.78,0.61) | -0.42 (-0.85,0.34) |
| D_7_ |  |  |  |  |  |  |
| JH | 0.06 (-0.63,0.70) | 0.62 (-0.07,0.91) | -0.51 (-0.88,0.23) | 0.37 (-0.37,0.79) | 0.87*(0.41,0.98) | -0.31 (-0.81,0.44) |
| RSI | 0.50 (-0.25,0.87) | 0.39 (-0.37,0.84) | -0.36 (-0.83,0.40) | 0.27 (-0.40,0.75) | 0.70*(0.00,0.94) | -0.47 (-0.86,0.29) |
| PF | 0.63 (-0.05,0.91) | 0.61 (-0.10,0.91) | -0.40 (-0.86,0.29) | 0.60 (0.00,0.88) | 0.90*(0.54,0.98) | -0.50 (-0.87,0.24) |
| PP | -0.38 (-0.83,0.38) | -0.15 (-0.74,0.57) | -0.50 (-0.87,0.25) | -0.60 (-0.88,0.00) | -0.24 (-0.81,0.56) | -0.50 (-0.87,0.24) |
| D_21_ |  |  |  |  |  |  |
| JH | 0.20 (-0.53,0.76) | 0.10 (-0.61,0.72) | -0.48 (-0.87,0.27) | 0.44(-0.22,0.82) | 0.58 (-0.21,0.91) | -0.26 (-0.79,0.49) |
| RSI | 0.60 (-0.10,0.91) | 0.63 (-0.06,0.91) | -0.32 (-0.81,0.43) | 0.27 (-0.39,0.75) | 0.90*(0.53,0.98) | -0.44 (-0.85,0.32) |
| PF | 0.61 (-0.09,0.91) | 0.48 (-0.27,0.87) | -0.45 (-0.86,0.30) | 0.58 (-0.03,0.87) | 0.86*(0.40,0.97) | -0.49 (-0.87,0.26) |
| PP | -0.32 (-0.81,0.44) | -0.21 (-0.77,0.52) | -0.36 (-0.83,0.40) | -0.62 (-0.89,-0.04) | -0.17 (-0.78,0.61) | -0.38 (-0.83,0.38) |
| \| Note. NC_1_=1st non-conference play. NC_2_=6th non-conference play. C_1_=1st conference-play. C_2_= 6th conference-play. D_2_= 2-day average. D_7_=7-day average. D_21_=21-day average. JH=Jump height. RSI=Modified reactive strength index. PF=Peak force. PP=Peak power. *=denotes *p*≤0.05 \| \| --- \| | | | | | | |
